# Effect of low-dose ketamine on PerioperAtive depreSsive Symptoms in patients undergoing Intracranial tumOr resectioN (PASSION): study protocol for a randomized controlled trial

**DOI:** 10.1186/s13063-018-2831-0

**Published:** 2018-08-29

**Authors:** Yang Zhou, Yuming Peng, Jinghan Fang, Wanchen Sun, Guofu Zhang, Long Zhen, Gang Wang, Ruquan Han

**Affiliations:** 1 Department of Anesthesiology, Beijing Tiantan Hospital, Capital Medical University, No. 6, Tiantan Xili, Dongcheng District, Beijing, 100050 People’s Republic of China; 2 China and Beijing Key Laboratory of Mental Disorders, National Clinical Research Center for Mental Disorders, Beijing Anding Hospital, Capital Medical University, Beijing, People’s Republic of China; 3 Department of Psychiatry, Capital Medical University, China and Center of Depression, Beijing Institute for Brain Disorders, Beijing, China

**Keywords:** Ketamine, Depression symptom, Perioperative, Randomized controlled trial

## Abstract

**Background:**

Perioperative depressive symptoms (PDS) are common mental comorbidities that influence clinical outcomes and prognosis. However, there is no rapid-acting treatment to address these symptoms during a limited hospital stay.

**Methods/design:**

This is a single-center, randomized, placebo-controlled, and double-blind trial. Randomization will be applied and stratified by the severity of PDS (moderate versus severe). Eighty patients who are scheduled for elective supratentorial brain tumor resection with PDS will be randomly allocated to the ketamine or placebo group with a ratio of 1 to 1. Patients in the ketamine group will be administered low-dose ketamine (0.5 mg/kg) intravenously for 40 min while the dural mater is being cut into, whereas patients in the placebo group will receive the same volume of normal saline at the same infusion rate at the same time points. The primary endpoint is the rate of PDS response at 3 days after surgery. Secondary outcomes include efficacy parameters such as the rate of PDS remission and safety outcomes such as the incidence of postoperative delirium, quality of recovery, and psychiatric side effects.

**Discussion:**

This study aims to determine whether ketamine could improve the depressive symptoms of perioperative patients undergoing supratentorial brain tumor resection. It will also examine the safety of administering ketamine as an intraoperative anti-depressant.

**Trial registration:**

ClinicalTrials.gov, NCT03086148. Registered on 22 March 2017.

**Electronic supplementary material:**

The online version of this article (10.1186/s13063-018-2831-0) contains supplementary material, which is available to authorized users.

## Background

Depression symptoms consist of mental problems that can be characterized by a loss of interest and enjoyment in daily life, low mood, and associated emotional, cognitive, physical, and behavioral symptoms [1]. Perioperative depressive symptoms (PDS) is one type of depressive episode that occurs during the perioperative period and has become a common perioperative complication. The prevalence of PDS in non-cardiac surgery patients was reported to be 24% [2], which is much higher than that observed in healthy people. Patients undergoing neurosurgery have an even higher rate, 44% [3, 4]. Depression symptoms deteriorate mental health and lead to poor clinical outcomes [5]. The incidence of suicide due to depression symptoms is reported to be increasing every year [6].

Pelletier et al. [7] conducted 6-month follow-up visits for 60 patients who had undergone brain tumor resection and found that the incidence of depression symptoms after surgery was nearly 38%, which seriously affected the quality of recovery from surgery. PDS is related to the severity of conditions in patients with brain tumors, such as the size, pathologic type, location, and mental state [7]. However, based on previous studies, depression symptoms before surgery suppress immune function and affect stress level and thyroid hormone levels [8], which consequently increase the incidence of perioperative cardiac events [9, 10], costs [11], and even suicide attempts during a hospital stay. Additionally, PDS increases the incidence of postoperative delirium, which leads to poor clinical outcomes [12, 13]. However, limited research has focused on the efficacious treatment of PDS in patients undergoing brain tumor resection.

Traditional anti-depressants are selective serotonin reuptake inhibitors (SSRIs) that block monoamine reuptake via the 5-hydroxytryptamine (5-HT) transporter and increase 5-HT in the synaptic space. Moreover, 5-HT regulates postsynaptic G protein-coupled receptors, which activate a variety of second messenger systems and up-regulate the expression of brain-derived neurotrophic factor (BDNF) in neurons [14, 15]. BDNF contributes to anti-depression by neuroprotection, neuroplasticity, and neurogenesis. However, the anti-depressive effect via SSRIs is a relatively slow process which needs more than 1 week to take effect [14, 15]. Hence, it is not feasible to treat patients with brain tumors who have PDS by administering traditional anti-depressants during their limited postoperative stay.

Ketamine is an *N*-methyl-d-aspartic acid receptor (NMDA) antagonist and is administered as both an analgesic and an anesthetic during surgery. Recent studies have indicated that ketamine has a rapidly anti-depressive effect. The Montgomery-Åsberg Depression Rating Scale (MADRS) and the Hamilton Depression Rating Scale are often used to evaluate the anti-depressive effect. In one study ketamine (0.5 mg/kg per body weight) was administered twice weekly for 2 weeks and improved depression with a response rate as high as 69% and a remission rate of 37.5% [16]. Fourteen studies enrolling 588 patients were reviewed [17], and low-dose ketamine (0.5 mg/kg) was found to significantly improve depression for 7 days. Li et al. [18] reported the same effect of ketamine in 675 patients undergoing electroconvulsive therapy. Additionally, the study of Lapidus et al. [19] noted that the anti-depressive effect of ketamine began to take effect 40 min after a single-dose administration and peaked at 24 h, with a remission rate of nearly 35% at 3 postoperative days. A consensus [20] was published in 2017 and recommended that low-dose ketamine (0.5 mg/kg) rapidly improved depression symptoms. Because of its quick action and significant anti-depressive effect, ketamine may be suitable to resolve PDS. However, there is no study on the effect of ketamine on PDS in patients undergoing surgery, especially brain tumor resection.

In addition, the mechanism of ketamine’s anti-depressive effects has not been clarified. Previous studies demonstrated that ketamine blocked the NMDA receptor, rapidly increased extracellular glutamate, and led to the activation of voltage-dependent calcium channels. With the calcium ion flux into cells, the downstream signaling pathways were stimulated and led to BDNF release [14, 15]. Ketamine shortened the time to express and release BDNF; thus, the onset time of ketamine may be shorter than those of other anti-depressants. Abdallah et al. [21] found that the concentration of BDNF in peripheral blood increased 72 h after administration of ketamine. However, some patients, such as those who carried the Met rs6265 allele, did not respond to ketamine [22]. Interleukin-6 (IL-6) was reported to be a biomarker for predicting the anti-depressive effects of ketamine [23]. The metabolites of ketamine were also found to be essential for its anti-depressive effects [24]. However, the anti-depression mechanism of ketamine is still under continual investigation.

In addition, safety is another critical issue when using ketamine to treat PDS. The side effects of ketamine include psychiatric symptoms, dizziness, nausea, and so on. All these side effects disappeared within 2 h after ketamine was administered intravenously [25], and the incidence of adverse events was reported to be very low and mild, especially with a low dose [26]. This outcome might be associated with the effective plasmatic concentration of anti-depressive in ketamine recommended ranging from 70 to 200 ng/ml, which is far below the peak plasma concentrations generally used as an anesthetic (2000–3000 ng/ml) [20]. Thus, using a low dose of ketamine to relieve PDS is theoretically safe for patients during surgery and general anesthesia. However, ketamine could increase the intracranial pressure (ICP) and cerebral metabolism and accelerate the recovery from general anesthesia [27]. It is still unknown whether intraoperative infusion of low-dose ketamine influences stable recovery from surgery and general anesthesia.

Based on the previous literature, we hypothesize that low-dose ketamine relieves PDS in patients undergoing supratentorial brain tumor resection. The primary endpoint is the response rate at postoperative 3 days, and the secondary endpoints include other efficacious parameters and the incidence of postoperative complications or side effects. We will conduct a randomized controlled trial to test this hypothesis.

## Methods/design

### Study design

This is a single-center, randomized, placebo-controlled, and double-blind trial. The patients will be screened and recruited consecutively in Beijing Tiantan Hospital, Capital Medical University. This trial will last for approximately one year and a half. Patients will be screened by two investigators.

### Study population

**Fig. 1:**
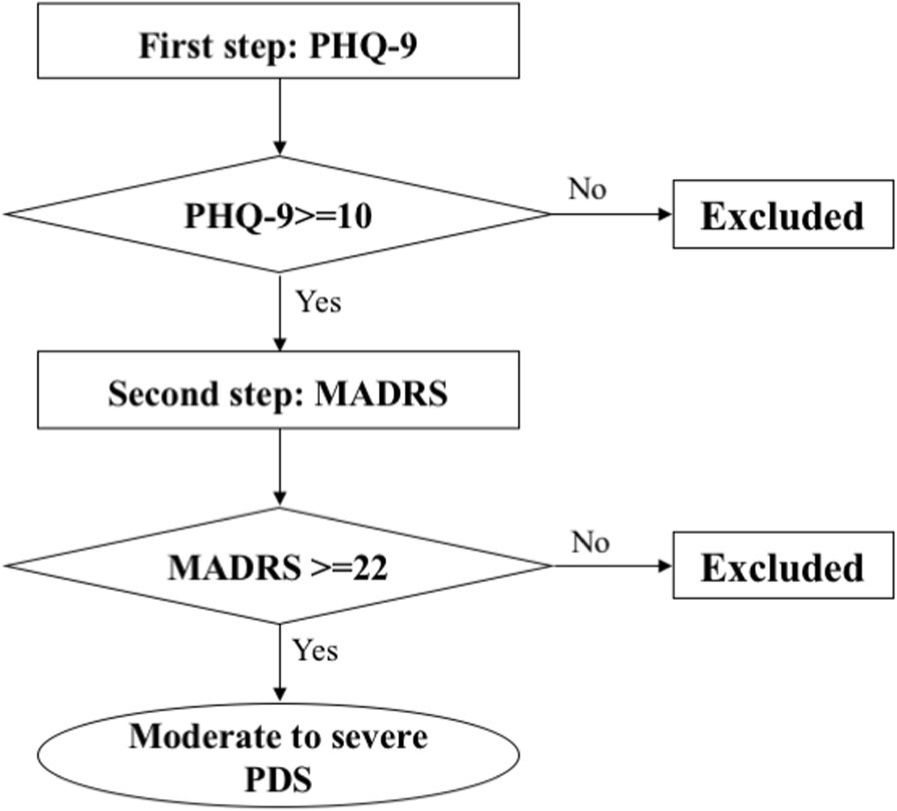
Flow chart of psychiatric assessment and diagnosis process. *PHQ-9* Patient Health Questionnaire-9, *MADRS* Montgomery-Åsberg Depression Rating Scale, *PDS* perioperative depressive symptoms

Patients with supratentorial brain tumors undergoing elective craniotomy resections will be screened for eligibility. The inclusion criteria will be as follows: an age range from 18 to 65 years old, having moderate to severe depressive symptoms, and an expected hospital stay of no less than 7 days. The depression will be evaluated through Patient Health Questionnaire-9 (PHQ-9) and MADRS, measured by trained and qualified psychiatric doctors 1 day before surgery. A score on the PHQ-9 of no less than 10 [28] and a score on the MADRS of no less than 22 are the required conditions to diagnose moderate to severe depressive symptoms. A flow chart of the psychiatric assessment and diagnosis process is presented in Fig. 1.

The exclusion criteria include the following: American Society of Anesthesiologists (ASA) physical classification status IV to V; history of epilepsy; major depressive disorder patients who have received anti-depressants within the past 2 weeks; psychiatric illness; drug abuse; history of allergy to the research drug; body mass index > 30 kg/m^2^; heart rate > 120 beats per minute; systolic blood pressure > 180 mmHg; heart failure; renal or liver dysfunction; tumor located in the Wernicke area, Broca area, or frontal pole; hyperthyroidism; patients who cannot cooperate to complete psychiatric assessments; pregnant or breast-feeding women; patients who refuse to sign informed consent.

### Randomization and blinding

Randomized blocks produced via computer will be conducted and stratified by moderate and severe PDS. Patients will be randomly assigned to the ketamine or placebo groups with a 1:1 ratio. The randomization list will be sealed in opaque envelopes and maintained by a person who will not participate in this study to guarantee allocation blinding.

Anesthesiologists, patients, and outcome assessors will be blinded to the group assignment until they complete the follow-up visit for the last enrolled participant. Both ketamine and placebo will be made into 50-ml volumes, and the ketamine concentration is 1 mg/ml. The investigated solution will be labeled as “trial solution” by a research assistant according to the randomization sequence. The assistant will not participate in anesthesia management, data collection, or any follow-up visit.

### Intervention

Patients will be randomly divided into the ketamine and placebo groups. In the ketamine group, ketamine will begin to be administered intravenously upon dural opening at a total dose of 0.5 mg/kg per body weight and will continue for 40 min. In the placebo group, the same volume of normal saline will be administered at the same infusion rate. All solutions will be infused intravenously at a speed of 0.75 ml kg^− 1^ h^− 1^ for 40 min. The investigated solutions will be administered by the chief anesthesiologist, who will be blinded to the grouping.

### Perioperative anesthesia management

Standard ASA parameters will be monitored perioperatively, including heart activity via electrocardiogram, non-invasive blood pressure, pulse oxygen saturation, body temperature, and bispectral index (BIS). Peripheral venous access and peripheral artery catheterization will be established before anesthesia induction. Continuous arterial pressure, urine output, and end-tidal carbon dioxide partial pressure (ETCO_2_) will be monitored and recorded.

After midazolam (0.05 mg/kg) is administered intravenously, anesthesia induction will be done with propofol (1–3 mg/kg) or etomidate (0.2–0.5 mg/ml), sufentanil (0.2–0.4 μg/kg), and rocuronium (0.6 mg/kg) or cisatracurium (0.2 mg/kg). Mechanical ventilation will be conducted with a tidal volume of 6–8 ml/kg, a respiratory frequency of 12–15/min, an inspiration and expiration ratio of 1:2, an inhaled oxygen fraction of 60%, and a fresh gas flow rate of 1–2 L/min to maintain the ETCO_2_ between 35 and 45 cmH_2_O. Remifentanil (0.1–0.3 μg kg^–1.^min^− 1^) and propofol (2–4 mg kg^–1^h^− 1^) or sevoflurane (1–3%) will be used to maintain the BIS between 35 and 50. Ondansetron (4–8 mg) will be given at the end of the surgery to prevent postoperative nausea and vomiting. Atropine and neostigmine will be administered to reverse the residual neuromuscular blockade.

Further, patient-controlled intravenous analgesia will be conducted routinely with sufentanil (background dose 0.02~ 0.04 μg kg^–1^ h^− 1^) and ondansetron (16 mg) diluted in 100 ml of normal saline. The pump will provide a basal infusion of 2 ml/h and bolus (0.5 ml, 15 min lock-out time).

Once the patient is assessed as having moderate to severe PDS, several additional measures will be taken including informing the chief nurse and doctors in the neurosurgical ward and providing a professional consultation from psychiatry as necessary.

### Outcomes

**Fig. 2:**
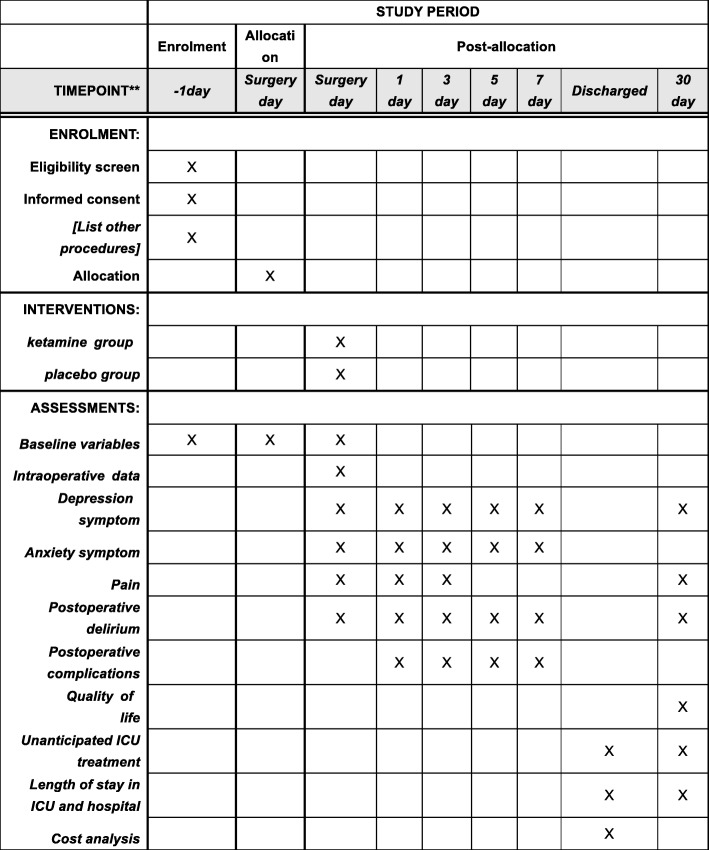
Standard Protocol Items: Recommendations for Interventional Trials (SPIRIT) figure showing the schedule of enrollment, intervention, and assessments

The aim of the trial is to observe the effect of low-dose ketamine on PDS in patients undergoing supratentorial brain tumor resection. The schedule of enrollment, intervention, and assessments is presented in Fig. 2 and Additional file 1.

The primary outcome is the rate of response to treating PDS at 3 postoperative days, which is defined as a relative reduction of more than 50% from the baseline 10-item MADRS score [29, 30]. The primary outcome as well as the other psychological assessment will be evaluated by the trained and qualified psychiatrist who is blinded to the grouping.

The secondary outcomes include the following efficacy and safety parameters:

1. The remission rate is defined as an absolute value of MADRS score of no more than 10 [31]. MADRS will also be assessed at 1, 2, 5, and 7 days after the administration of ketamine.
2. Perioperative anxiety symptoms will be assessed by the Hospital Anxiety and Depression Scale (HADS) at 1, 2, 3, 5, and 7 days after administration. Anxiety symptoms will be defined as a HADS score of no less than 11 [32].
3. Postoperative delirium will be assessed by the Confusion Assessment Method for the Intensive Care Unit Scale (CAM-ICU) at 2 h and 1 day after surgery [33].
4. The incidence of severe pain will be assessed within the first 3 postoperative days and defined using the mean and peak numerical rating scale (NRS) with a score higher than 5 [34].
5. Intraoperative awareness will be screened by the modified Brice questionnaire 1 day after surgery [35].
6. Postoperative complications will be recorded, including psychiatric symptoms evaluated by the Brief Psychiatric Rating Scale (BPRS), manic symptoms assessed by the 11-item Young Mania Rating Scale (YMRS), and dissociative symptoms measured by the Clinician Administered Dissociative States Scale (CADSS) [36]. Moreover, postoperative complications will include deep sedation, euphoria, hallucination, pathological dysphoria, nightmares, and sleeplessness.
7. The length of stay and cost of the ICU and hospital will be determined.
8. The quality of life will be evaluated by Karnofsky Performance Status (KPS) at postoperative 30 days [37].
9. The plasmatic concentrations of ketamine, its metabolites, and cytokines (including BDNF, IL-6, and d-cycloserine) will be tested at different time points after ketamine is administered [38].

### Sample size calculation

Previous studies reported that nearly 44% of all patients with brain tumors experience PDS [39]. Patients with moderate to severe depression symptoms, as screened by PHQ-9 and MADRS, will be enrolled in this study. It has been reported that depression patients treated with ketamine showed a response rate of 53.8% [30]. We conservatively assume a response rate of 40% in the ketamine group and 10% in the placebo group at postoperative day 3. Thus, the effect size will be 30% for the response rate. Given an alpha level of 0.05, a beta of 0.2, and an additional dropout rate of 5%, the total sample size required is 80 (40 in each group). Based on our pre-study survey, the rate of moderate to severe PDS in patients with brain tumors is approximately 7.91%. Therefore, nearly 1000 patients will be screened for inclusion in the study.

### Statistical analysis

The normally distributed measurement data will be reported as the mean with standard deviation, skewed data will be reported as the median with interquartile range, and categorical data will be described by the count (percentage). Continuous outcomes will be analyzed by the Kolmogorov-Smirnov test to judge normality. The between-group differences in all endpoints will be compared by using Student’s *t* test for normally distributed variables, the Mann-Whitney *U* test for skewed variables, and the chi-square test for categorical variables.

For the primary endpoint of the response rate, we will use the chi-square test to compare the between-group differences. The incidence of anxiety and delirium will be compared by using the chi-square test. The analysis of variance (ANOVA) for repeated measurements will be employed to detect the differences in repeated measurement data between the groups (e.g., MADRS, CAM-ICU, NRS, BPRS, YMRS, CADSS, and opioid consumption). The incidence of adverse events (such as nightmares, nausea, and vomiting) will be analyzed by a chi-square test. The length of hospital or ICU stay and the postoperative 30-day KPS score will be investigated for the differences between groups by dependent *t* test.

In order to delete the effect of confounding factors and covariates, we will establish a multivariate logistics regression model to observe the effect of a small dose of ketamine on PDS in patients undergoing supratentorial brain tumor resections. The potential variables in the model include the strata (the severity of preoperative depression), gender, income level, education degree, neurological function (KPS, Charlson comorbidity index) and tumor characteristics (type, size, and site). The impact of missing data will be estimated by sensitivity analysis. A two-sided *P* value less than 0.05 will be considered significant. The Stata 14.0 software (Stata Corp LP, College Station, TX, USA) will be used.

### Reporting of adverse events

All adverse events associated with this trial will be closely monitored until they are resolved and stable. Once an adverse event occurs, it will be immediately reported to the department, and the principal investigator will be informed to determine the severity of the adverse event and the consequence. All adverse events associated with this study will be recorded and reported to the Ethics Committee within 1 week, which will be part of the annual report. The principal investigator will be responsible for all reported adverse events.

## Discussion

The PASSION is a single-center, randomized and placebo-controlled trial that aims to explore the effect of low-dose ketamine on PDS in patients undergoing supratentorial brain tumor resection. The patients will be intravenously administered ketamine (0.5 mg/kg) for 40 min when the dural mater is opened. The efficacy and safety issues concerning the effect of ketamine on PDS in patients with brain tumors will be tested.

PDS was indicated to be one of the risk factors for postoperative delirium and affected consciousness during recovery periods [13]. Several clinical trials suggested that ketamine reduced the incidence of delirium and postoperative pain during the recovery period. However, Avidan and colleagues [40] found that ketamine failed to reduce delirium or postoperative pain but instead increased the negative experiences. The wound pain after surgery might trigger several postoperative complications [34] including depression symptoms. Recent studies have found a correlation between pain and depression [41]. Ketamine has a strong analgesic effect [42, 43]. However, whether its anti-depressive effect is based on analgesia or its inherent attributes remains unclear. Hence, the confounding effect of postoperative delirium and pain should also be evaluated when the effect of ketamine on PDS is studied.

A low dose (0.5 mg/kg) of ketamine has often been used to address treatment-resistant depression. We set 0.5 mg/kg as the interventional dose, based mainly on the consensus for applying ketamine in mood disorders [20]. In addition, considering the requirement of coordination with postoperative psychiatric assessments, we will use postoperative 3 days as the primary time point for the primary outcome. Furthermore, the investigators will screen patients before surgery to determine whether their MADRS score is more than 22, which indicates moderate depression. Thus, the absolute and relative changes in the MADRS score from baseline will be suitable and accurate to explore the anti-depressive effects of ketamine and are defined as the remission and response rates, respectively [16–20, 31].

The issue of safety when using ketamine to treat PDS includes increasing ICP, psychiatric symptoms, and addiction. To avoid having an impact on ICP, ketamine will be given upon dural opening, when ICP has already reached zero. In addition, the plasma concentration after 0.5 mg/kg ketamine administered was approximately 70–200 ng/ml, which was significantly lower than the anesthetic plasmatic concentration (2000~ 3000 ng/ml) [20], and no severe psychiatric symptoms were reported under the low dose of ketamine [26]. Psychiatric symptoms and somatization disappeared 2 h after the ketamine infusion was stopped. In the current trial, the low-dose ketamine was administered only once, and its effects lasted for approximately 40 min, which can help avoid transient high peak plasma concentrations from bolus [20]. However, we should still pay more attention to the recovery quality from anesthesia, which may be influenced by ketamine.

In recent studies on ketamine, the control group always set normal saline or midazolam as placebo [44]. In the current trial, the reasons for why we use normal saline as the comparator as follows: First, PDS in subjects with brain tumors are no more severe than those in patients with refractory depression. Second, there is no anti-depressant that begins to take effect in less than 1 week, but patients undergoing brain tumor resection are often discharged within 7–10 days. Finally, it seems unnecessary to set an active comparison under general anesthesia. However, to ensure the safety of participants enrolled with moderate to severe depression, we will inform the chief nurse and doctors in the neurosurgical ward and apply a professional consultation from psychiatry as necessary.

In summary, the study is a randomized, controlled, and double-blind trial aiming to observe the effect of low-dose ketamine on PDS in patients with brain tumors. The expected result is that ketamine could markedly and safely relieve PDS in patients undergoing supratentorial tumor resection. This trial will also bring strong focus on patients with perioperative mental health issues and explore measures to improve prognosis.

### Trial status

The trial was registered at ClinicalTrials.gov on 22 March 2017 (identifier NCT03086148). The study was approved by the Institutional Review Boards at Beijing Tiantan Hospital, Capital Medical University on 23 May 2017 (reference number KY2017-023-02). The first participant was recruited on 5 July 2017, and the anticipated completion date will be in December 2018.

## Additional file

Additional file 1: SPIRIT 2013 checklist: recommended items to address in a clinical trial protocol and related documents. (DOC 136 kb)

## Abbreviations

5-HT: 5-hydroxytryptamine
BDNF: Brain-derived neurotrophic factor
BIS: Bispectral index
BPRS: Brief Psychiatric Rating Scale
CADSS: Clinician Administered Dissociative States Scale
CAM-ICU: Confusion Assessment Method for the Intensive Care Unit Scale
ETCO_2_: End-tidal carbon dioxide partial pressure
HADS: Hospital Anxiety and Depression Scale
ICP: Intracranial pressure
IL-6: Interleukin-6
KPS: Karnofsky Performance Status
MADRS: Montgomery-Åsberg Depression Rating Scale
NIHSS: National Institute of Health Stroke Scale
NMDA: *N*-methyl-d-aspartic acid receptor
NRS: Numerical rating scale
PDS: Perioperative depressive symptoms
PHQ-9: Patient Health Questionnaire-9
RCT: Randomized controlled trial
SSRI: Selective serotonin reuptake inhibitor
YMRS: Young Mania Rating Scale
